# Safe digital isolation of the santorini plexus during radical retropubic prostatectomy

**DOI:** 10.1186/1471-2490-13-13

**Published:** 2013-02-27

**Authors:** Cristiano Cristini, Giovanni Battista Di Pierro, Costantino Leonardo, Cosimo De Nunzio, Giorgio Franco

**Affiliations:** 1 Department of Gynaecological-Obstetrical and Urological Sciences, ‘Sapienza’ University, Rome, Italy

**Keywords:** Digital isolation, Dorsal vein complex, Radical retropubic prostatectomy, Santorini plexus

## Abstract

**Background:**

We describe a safe and easily reproducible technique to control Santorini plexus during radical retropubic prostatectomy (RRP) which uses simple digital dissection.

**Methods:**

We retrospectively reviewed 56 consecutive patients who underwent RRP for clinically localised prostate cancer from November 2008 to November 2010. Santorini plexus was isolated and secured in all patients using a new technique of simple digital dissection in which the index finger is used not to only localize the catheter inside the urethra, but also to develop the right plane between Santorini plexus and urethra. This is obtained by gentle bilateral digital dissection through the lateral aspects of periprostatic fascia which are eventually breached by the fingers, developing a right plane just above the urethra. Santorini plexus is then easily ligated and divided. Indicators of outcomes included estimated blood loss, transfusion requirements, operative time, positive margins and complication rates of the technique.

**Results:**

The maneuver was successful in 53/56 (95%) patients. Mean (range) blood loss and overall operative time for RRP were 620 ml (100–1500) and 130 min. (80–190), respectively. Transfusion rate was 8,9% (5/56). Positive surgical margin rate was 14% (8/56). No complication related to the employed technique was recorded.

**Conclusions:**

Digital dissection of Santorini plexus during RRP is simple and easily feasible. It speeds up the process of finding the right plane just above the urethra allowing good haemostasis in the surgical field and proper apical dissection.

## Background

Prostate cancer is the most frequently diagnosed cancer and cause of cancer-related death in men. Since introduction, retropubic radical prostatectomy (RRP) has been considered as gold standard surgical treatment for localised prostatic cancer [1].

Although the use of minimally invasive techniques has increased in recent years, RRP still represents the most frequently employed surgical treatment in most countries [2].

Control of the dorsal vein complex (DVC) or Santorini plexus remains one of the most challenging steps of the procedure to reduce blood loss, avoid damage to the sphincteric muscle and maximize the chance of cure. It is often accomplished by blindly passing a clamp between urethra and DVC before ligation and division [3]. Over the years, several approaches have been developed. In a study by Rainwater and Segura sutured control of the DVC before transection resulted in a significant decrease in the estimated blood loss than unsutured approach [4]. Subsequently, various techniques have been reported including combined infrapubic and retropubic ligation [5], suture passers [6,7], surgical staplers [8,9] or Babcock clamp use before ligation and transection [10]. However, management of DVC does not always result easy as well as sometimes difficulties in introducing the clamp exactly in the right plane may be encountered, so that it passes too superficially, inside the Santorini plexus, or too deeply damaging the urethra and the sphincteric muscle. In 2009 Namiki et al. [11] described a novel technique using careful digital dissection to better delineate the apex of the prostate in order to lessen positive surgical margins at the apex and promote continence and erectile function.

## Methods

### Technique

According to Walsh’s technique [1], after removing the fatty tissue surrounding the DVC and incising the endopelvic fascia on both sides of the prostate, the index finger is used to gently separate the levator ani muscle from the prostate. The guidance of index finger feeling the catheter allows for the clamp passage just above the urethra, blindly perforating the lateral fascia and thus separating the urethra from the Santorini plexus.

**Figure 1:**
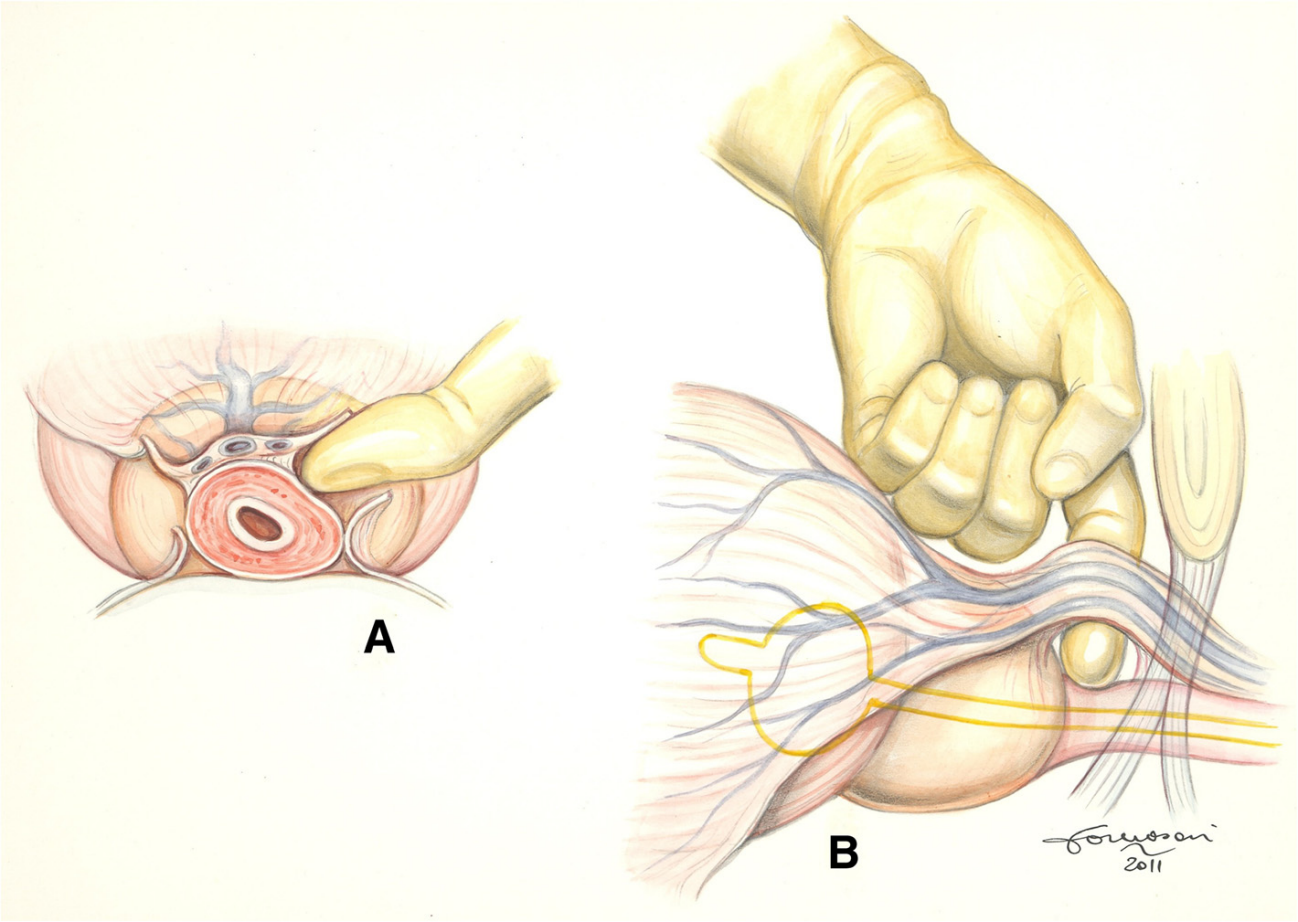
**A) Axial view.** The left index finger has breached the left lateral aspect of the periprostatic fascia containing the DVC and the urethra and is separating the two structures. The right lateral aspect of the fascia is depicted already breached (by the right index finger). **B**) Lateral view. The right plane between the DVC and the urethra with its rabdosphincter has been fully developed.

**Figure 2:**
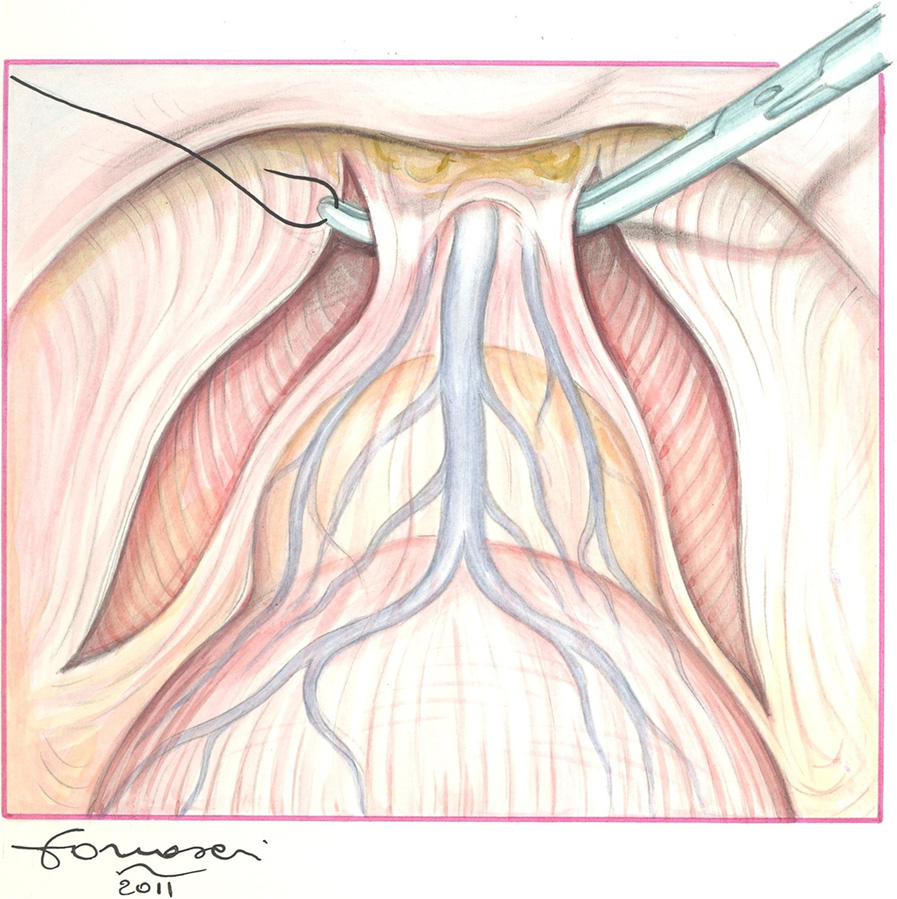
**Frontal view.** A suture can now be passed in order to secure and divide the DVC.

In our modified technique, the finger is not only used to localise the urethra, but also to develop the right plane between Santorini plexus and urethra with the finger tip forced just above this structure. The fascia is eventually breached sequentially on both sides, first the right one with the right index finger and then the left one with the left index finger of the surgeon (Figure 1A-B). This plane is easily followed with minimal bleeding and few chances of involving the anterior urethral wall. Once the right and left index finger tips are able to rendez-vous below the isolated Santorini plexus, it is easy and safe to pass a right–angled clamp for ligation (Figure 2) or direct division of DVC. Procedure then follows the classical steps of retrograde radical retropubic prostatectomy.

### Patient cohort and measurements

After approval from the institutional review board (University of Rome), we reviewed, from November 2008 to November 2010, 56 consecutive patients diagnosed with organ-confined prostate cancer who underwent RRP at our institution. Patient mean (range) age was 65 years (48.0-73.0) and PSA 7 ng/ml [2-20]. None had preoperative radiotherapy and/or neoadjuvant androgen deprivation therapy. Autologous blood donation was not routinely recommended preoperatively. All patients had given written informed consent and RRP was carried out by 2 surgeons (C.C. and G.F.).

Indicators of outcomes included: estimated blood loss, transfusion requirements, operative time, positive margins and complication rates of the technique.

### Utility

We describe an easily reproducible technique which, similarly to the procedure described by Namiki et al., allows securing of DVC during RRP by the simple digital development of the right plane between urethra and DVC allowing an easy and safe clamp passage for its ligation.

## Results

Demographics, clinical and pathological characteristics of patients are listed in table (Table 1). The maneuver was successful in 53/56 (95%) patients. In three patients it was not possible to breach the fascia with the finger due to intensive fibrosis and the standard technique of blind passage of a clamp was employed. Mean (range) blood loss and overall operative time for RRP were 620 ml (100–1500) and 130 min. (80–190), respectively. Transfusion rate was 8,9% (5/56). Positive surgical margin rate was 14% (8/56): 1 pT2c, 4 pT3a and 3 pT3b. No complication related to the employed technique was recorded. Continence (no leakage at all) and potency (IIEF-5 > 20) rates were assessed at minimum one year of follow-up (range 12–34 months) and were 88% and 41% respectively.

**Table 1:** Patient characteristics

	
**Age, yr, median (IQR)**	**65 (48.0-73.0)**
**PSA level, ng/ml, median (IQR)**	**7.2 (2.1-20.3)**
**Biopsy Gleason Score, n.**	
**6**	**12 (22%)**
**7**	**30 (53%)**
**8-10**	**14 (25%)**
**Clinical Stage, n.**	
**cT1c**	**25 (44.8%)**
**cT2a-b**	**30 (53.7%)**
**cT3**	**1 (1.5%)**
**Pathological Gleason Score, n.**	
**6**	**2 (3.5%)**
**7**	**44 (78.5%)**
**8-10**	**10 (18.0%)**
**Pathological Stage, n.**	
**cT1c**	**42 (74.7%)**
**cT2a-b**	**13 (23.1%)**
**cT3**	**1 (2.2%)**
**Pathological positive lymph node, n.**	**9 (16%)**
**Positive surgical margins, n.**	**8 (14%)**

*IQR* = Interquartile range.

## Discussion

Management of DVC is an important step of radical prostatectomy. In 1979 Reiner and Walsh described an anatomical approach to control this plexus [3].

Aside from reduction in blood loss, precise understanding of DVC anatomy have also resulted in a better apical dissection and preservation of the neurovascular bundles at this level, allowing improved continence and potency rates [6,12,13]. However, intraoperative bleeding still remains an issue in contemporary RRP [14,15] and any possible measure helpful in reducing should be taken into account. In light of this, pure laparoscopic and then robot-assisted techniques have been also introduced to reduce bleeding mainly exploiting the compressive effect of the pneumoperitoneum. As in LRP and RALP different techniques have been assessed to handle DVC [16-18], also in RRP there is a need for more effective ways to obtain haemostasis.

Another important aspect to consider is the postprostatectomy incontinence: avoiding damage to the rhabdosphincter is fundamental. Indeed, use of coagulation for hemostasis or not careful ligation before transection may damage the rhabdosphincter and underlying neurovascular components [19,20]. The blunt digital apical dissection technique described by Namiki et al. represents a clever way to better delineate the apex of the prostate in order to improve results in terms of positive surgical margins, continence and erectile function. Similarly, our technique exploits the digital dissection of the DVC from the urethra, but distally to the prostatic apex, with the main aim to safely and quickly develop a right plane between the two structures. In this way, a safer and easier way to control Santorini plexus while performing RRP is provided. This study suggests its feasibility in order to obtain sufficient haemostasis for a better apical dissection before the prostate had been mobilized and removed. Our results confirms the advantages of the technique described by Namiki et al., and supports its routinary use during RRP.

Limitations of the present study are its retrospective and small cohort analysis without a comparison with our standard technique of blind clamp passage between urethra and DVC.

Finally, since this is only an initial experience, further evaluation is necessary to determine the impact of this technical modification on functional and oncological outcomes. As the follow-up matures, we will be able to report.

## Conclusions

The described technique allows for a safe and easily reproducible isolation of the Santorini plexus during radical retropubic prostatectomy. We believe that using the finger, our most clever surgical instrument, we can make this procedure safer and more approachable particularly in the hands of the less experienced surgeon.

## Competing interests

The authors declare that they have no competing interests.

## Authors' contributions

GF, contributions to conception and design, GdP: acquisition of data and interpretation of data, CL: drafting the manuscript, CdN: revising the manuscript critically for important intellectual content, CC: final approval of the version to be published. All authors read and approved the final manuscript.

## Pre-publication history

The pre-publication history for this paper can be accessed here:

http://www.biomedcentral.com/1471-2490/13/13/prepub
